# Assessment of the efficacy and toxicity of ^131^I-metaiodobenzylguanidine therapy for metastatic neuroendocrine tumours

**DOI:** 10.1038/sj.bjc.6604273

**Published:** 2008-02-19

**Authors:** A C Nwosu, L Jones, J Vora, G J Poston, S Vinjamuri, D M Pritchard

**Affiliations:** 1 Division of Gastroenterology, University of Liverpool, Liverpool, UK; 2 Centre for Digestive Diseases, Aintree University Hospitals NHS Trust, Liverpool, UK; 3 Department of Diabetes and Endocrinology, Royal Liverpool and Broadgreen University Hospitals NHS Trust, Liverpool, UK; 4 Department of Nuclear Medicine, Royal Liverpool and Broadgreen University Hospitals NHS Trust, Liverpool, UK

**Keywords:** Carcinoid, neuroendocrine, radionuclide, ^131^I-MIBG

## Abstract

^131^I-metaiodobenzylguanidine (^131^I-MIBG) is a licensed palliative treatment for patients with metastatic neuroendocrine tumours. We have retrospectively assessed the consequences of ^131^I-MIBG therapy in 48 patients (30 gastroenteropancreatic, 6 pulmonary, 12 unknown primary site) with metastatic neuroendocrine tumours attending Royal Liverpool University Hospital between 1996 and 2006. Mean age at diagnosis was 57.6 years (range 34–81). ^131^I-MIBG was administered on 88 occasions (mean 1.8 treatments, range 1–4). Twenty-nine patients had biochemical markers measured before and after ^131^I-MIBG, of whom 11 (36.7%) showed >50% reduction in levels post-therapy. Forty patients had radiological investigations performed after ^131^I-MIBG, of whom 11(27.5%) showed reduction in tumour size post-therapy. Twenty-seven (56.3%) patients reported improved symptoms after ^131^I-MIBG therapy. Kaplan–Meier analysis showed significantly increased survival (*P*=0.01) from the date of first ^131^I-MIBG in patients who reported symptomatic benefit from therapy. Patients with biochemical and radiological responses did not show any statistically significant alteration in survival compared to non-responders. Eleven (22.9%) patients required hospitalisation as a consequence of complications, mostly due to mild bone marrow suppression. ^131^I-MIBG therefore improved symptoms in more than half of the patients with metastatic neuroendocrine tumours and survival was increased in those patients who reported a symptomatic response to therapy.

Neuroendocrine tumours (NETs) are rare and their aetiology is poorly understood. Many tumours grow slowly, although some can grow very rapidly (Modlin *et al*, 2003, 2005). They express neuroendocrine differentiation markers, have secretory characteristics and may present with hypersecretory syndromes (Plockinger *et al*, 2004; Ramage *et al*, 2005). Unlike patients with adenocarcinomas at the same sites, patients with NETs tend to experience a longer indolent duration of their condition. Although the incidence of NETs is low (3–4 cases per 100 000 per annum) (Ramage *et al*, 2005), the good prognosis of many with these tumours has resulted in an increasing prevalence.

Many patients present when their tumour has already metastasised. In such patients, alleviation of hormonal symptoms and prolongation of survival are key goals (Plockinger *et al*, 2004; Ramage *et al*, 2005). Palliative treatment options include surgical resection of the primary tumour if symptomatic, treatment of liver metastases (by debulking surgery, hepatic artery embolisation or radiofrequency ablation), chemotherapy, interferon-*α*, somatostatin analogues and radionuclide therapies (Plockinger *et al*, 2004; Ramage *et al*, 2005).

^131^I-metaiodobenzylguanidine (^131^I-MIBG) is a licensed systemic treatment for the palliation of patients with metastatic NETs. ^131^I-MIBG is a guanethidine derivative, structurally similar to noradrenaline and thus is accumulated in tissues arising from neural crest cells (Wafelman *et al*, 1994). ^131^I-MIBG is transported by vesicular monoamine proteins and is concentrated in intracellular storage vesicles (Kolby *et al*, 2003). It is thought that emission of ionising radiation at this site results in tumour decay (Mukherjee *et al*, 2001; Rose *et al*, 2003; Pasieka *et al*, 2004). This therapy has been used to treat NETs of various types, including gastroenteropancreatic NETs (Pathirana *et al*, 2001; Pasieka *et al*, 2004; Safford *et al*, 2004; Sywak *et al*, 2004; Buscombe *et al*, 2005), paraglangliomas (Mukherjee *et al*, 2001; Safford *et al*, 2003; Fitzgerald *et al*, 2006), phaeochromocytomas (Castellani *et al*, 2000; Rose *et al*, 2003; Safford *et al*, 2004; Fitzgerald *et al*, 2006), medullary carcinoma of the thyroid (Castellani *et al*, 2000, 2003; Mukherjee *et al*, 2001) and neuroblastomas (Howard *et al*, 2005; Matthay *et al*, 2007). A number of previous studies had demonstrated significant symptomatic benefit in 40–60% of patients with metastatic NETs following ^131^I-MIBG therapy (Safford *et al*, 2004; Sywak *et al*, 2004), in a safe and cost-effective manner (Pathirana *et al*, 2001).

Few studies have, however, investigated whether ^131^I-MIBG also increases the survival of patients with metastatic NETs. We have therefore assessed the efficacy and toxicity of ^131^I-MIBG therapy in a 10-year cohort of patients with metastatic NETs.

## METHODS

### Patients

A total of 58 patients were identified from the records of the Nuclear Medicine department of Royal Liverpool and Broadgreen University Hospitals NHS Trust as having received ^131^I-MIBG therapy between May 1996 and May 2006. The study was registered at the hospital audit department and approval was obtained to perform the study. All patients were selected for ^131^I-MIBG therapy following discussion at a multidisciplinary meeting when the disease was metastatic, patients were symptomatic, curative surgical treatment was not possible and patients showed strong tumour uptake of ^123^I-MIBG by scintigraphy. Additional treatments were considered on an individual basis following discussion at the multidisciplinary team meeting.

All case notes were obtained and a retrospective case note analysis was performed. The following parameters were recorded for each case: patient demographics; date of diagnosis; primary site of tumour, site of metastases and details of histology; previous treatments administered; blood and urine results (including full blood count, serum electrolytes, liver function tests, serum chromogranin A, fasting gut hormone profile, 24 h urinary 5HIAA concentration) and results of all imaging studies performed (including computerised tomography (CT), ultrasound, magnetic resonance imaging (MRI), ^111^In-octreotide and ^123^I-MIBG scans). In addition, the dates of all ^131^I-MIBG therapies were recorded and details of all blood tests and imaging studies performed post-therapy to monitor the side effects and therapeutic effects of each treatment cycle were kept.

Of the 58 patients who received ^131^I-MIBG therapy, 48 patients were identified as having metastatic gastroenteropancreatic or pulmonary NETs and these individuals were included in the study. The remainder had other tumour types (mostly paraganglioma, phaeochromocytoma, and medullary carcinoma of the thyroid) and were excluded from further analysis. Neuroendocrine tumours were diagnosed in 33 (68.8%) cases on the basis of confirmatory histology, but in 15 (31.2%) cases no biopsy was performed and the diagnosis was established on the basis of positive imaging studies (such as CT, ^123^I-MIBG or ^111^In-Octreotide scans), along with significantly elevated titres of biochemical markers (24 h urinary 5HIAA and/or serum chromogranin A) in patients presenting with symptoms compatible with the carcinoid syndrome.

### Analysis of response to treatment

All patients received 7.4 GBq ^131^I-MIBG for each treatment cycle, and thyroid blockade was given using 170 mg of potassium iodate per day per treatment cycle. Response to ^131^I-MIBG therapy was assessed using standard World Health Organization (WHO) criteria in a similar manner to previous studies (Safford *et al*, 2004). A course of treatment was considered as the completion of therapy from the first to the last treatment of ^131^I-MIBG. Symptomatic responders were defined as patients reporting either complete resolution or a subjective decrease in the intensity of symptoms. These symptoms included abdominal pain, flushing, bloating, diarrhoea, weight loss, fatigue, sweats, breathlessness, vomiting, haemoptysis and anorexia. Patients were defined as biochemical responders if serum chromogranin A and/or 24 h urinary 5HIAA levels showed either complete normalisation or a reduction of >50% in previously elevated concentrations. A radiological tumour response was defined as either complete regression of tumour burden or a >25% reduction in tumour size based on analysis of post-therapy ^131^I-MIBG, CT or MRI scans by a consultant radiologist. The NCI common toxicity criteria were used to assess the toxicity of ^131^I-MIBG therapy. Toxicity was assessed by monitoring full blood count, serum electrolytes and thyroid function tests after each dose of ^131^I-MIBG.

### Statistical analysis

Data were stored and analyzed using the Statistical Software Package for the Social Sciences (SPSS version 14.0). Survival is quoted as median±s.e. Survival outcomes were plotted using the Kaplan–Meier method. *χ*^2^ tests (Log-rank, Wilcoxon and Tarone–Ware) were used to compare categorical variables. Statistical significance was set at *P*<0.05.

## RESULTS

### Patient characteristics

**Figure 1:**
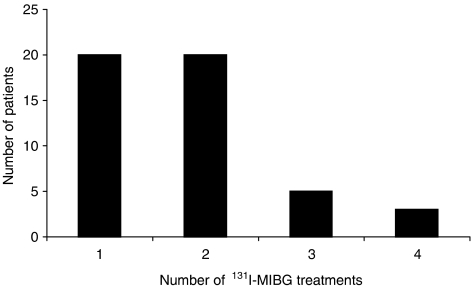
Number of patients receiving 1, 2, 3 or 4 cycles of ^131^I-MIBG therapy. Total patient number is 48. Mean number of treatments per patient was 1.8.

A total of 48 patients (21 male, 27 female) were identified as having received ^131^I-MIBG therapy for NETs (30 gastroenteropancreatic, 6 pulmonary, 12 with unknown site of primary tumour) (Table 1). The mean age at diagnosis was 57.6 years (range 34–81). All patients were identified as having metastatic disease, with liver metastases being present in 44 (91.7%). The most common presenting symptoms were diarrhoea, flushing and abdominal pain (present in 60.4, 58.3 and 41.7% of patients, respectively) (Table 1). Twenty-nine (60.4%) patients had received surgery at some point as part of the management of their condition. Small bowel resection was the most common procedure, being performed in 13 (27.1%) cases. Thirty-seven (77.1%) patients received short or long acting somatostatin analogues at some stage during the course of their treatment. Although many patients experienced some benefit from these agents, they all had residual symptoms and the responses described below to ^131^I-MIBG therapy, all occurred while patients remained on a stable dose of somatostatin analogues. Six (12.5%) patients had also received chemotherapy, two (4.2%) interferon-*α*, one (2.1%) radiofrequency ablation of liver metastases and seven (14.6%) hepatic chemoembolisation at some point during the course of their illness. ^131^I-MIBG was administered to these patients on 88 occasions (mean 1.8 treatments per patient, range 1–4) (Figure 1). The median time from initial diagnosis to first ^131^I-MIBG treatment was 38.5±9.5 months and the median follow up post-^131^I-MIBG treatment was 31.0±3.5 months (range 7–127 months).

### Response to ^131^I-MIBG therapy

Median survival for the 48 patients was 79±7.2 months from initial diagnosis and 46±4.6 months from first ^131^I-MIBG treatment. Of the 26 patients who died during the study period, median time from initial diagnosis to death was 57.5±6.3 months and median time from first ^131^I-MIBG treatment to death was 19.5±4.7 months.

Twenty-nine patients were identified in whom records of biochemical tumour marker measurements performed before and after ^131^I-MIBG therapy could be found. These investigations were performed while patients continued to take a stable dose of somatostatin analogue, if they were prescribed such therapy. Of these 29 patients, 11 (36.7%) showed >50% reduction in concentrations of serum chromogranin A and/or 24 h urinary 5HIAA post-^131^I-MIBG therapy (Table 2). Similarly, 40 patients were identified in whom reports were available for analysis of radiological investigations performed before and after ^131^I-MIBG therapy. Eleven (27.5%) of these patients showed >25% reduction of tumour size on post-therapy scans (Table 2). Records of symptomatic responses to ^131^I-MIBG therapy were available for all 48 patients from follow up outpatient clinic letters. Twenty-seven (56.3%) patients reported improvement in their symptoms following ^131^I-MIBG therapy (Table 2).

**Figure 2:**
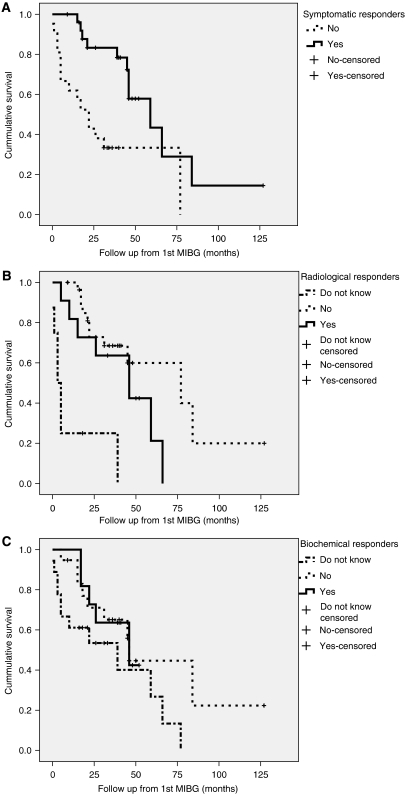
Kaplan–Meier charts demonstrating patient survival from the date of first ^131^I-MIBG treatment for (**A**) symptomatic, (**B**) radiological and (**C**) biochemical variables comparing responders to ^131^I-MIBG therapy with non-responders.

Kaplan–Meier analysis showed significantly increased survival (59.0±13.9 months) from the date of first ^131^I-MIBG treatment in those patients who reported symptomatic benefit following therapy compared to those who showed no improvement in symptoms (22.0±5.3 months) (*P*<0.01; Figure 2A). Patients in whom results of radiological scans post-^131^I-MIBG therapy could not be found survived for shorter periods of time (median 3.0±1.4 months) compared to those with in whom the results of radiological follow up scans post-^131^I-MIBG (median 59±7.9 months) (*P*<0.001) were available. This may be because the former group of patients died before follow-up scans could be arranged. However, there was no significant difference in survival between those patients who showed improvement in radiological scans (median 46±14.7 months) compared with those who showed no change or progression on radiological scans (median 77±28.8 months) (Figure 2B). Patients identified as biochemical responders also did not show any significant difference in survival compared to non-responders (median survival 46.0±18.7 months in biochemical responders compared to 46.0±1.3 months in non-responders) (Figure 2C).

Tumours were subgrouped into those arising in the gastrointestinal tract, pancreas, lung and unknown primary site to assess whether tumour site affected response to ^131^I-MIBG therapy. There was no significant correlation between tumour site and either patient survival or symptomatic, radiological or biochemical response to ^131^I-MIBG therapy.

In addition, there were no significant differences between those patients having liver resection (six patients), radiofrequency ablation (one patient) or chemoembolisation (seven patients) of liver metastases and the remainder of the cohort who did not have cytoreductive liver treatment in terms of symptomatic response or survival following ^131^I-MIBG therapy.

Although there was a trend towards more courses of ^131^I-MIBG therapy being administered to symptomatic responders (mean 2.07 treatments) compared to symptomatic non-responders (mean 1.48 treatments), this difference was not statistically significant. Similarly, there were no statistically significant differences in the number of ^131^I-MIBG treatments administered to radiological responders (mean 1.73 treatments) *vs* non-responders (mean two treatments) and biochemical responders (mean 1.91 treatments) *vs* non-responders (1.86 treatments).

**Figure 3:**
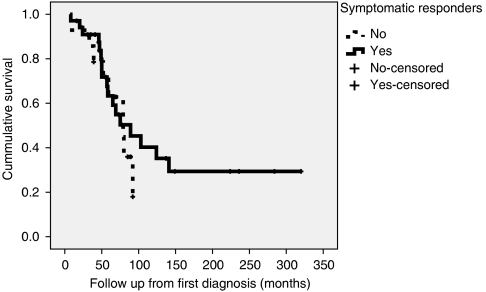
Kaplan–Meier chart demonstrating patient survival from the date of diagnosis in symptomatic responders to ^131^I-MIBG therapy compared with non-responders.

Although symptomatic responders demonstrated significantly increased survival compared to non-responders from the date of first administration of ^131^I-MIBG therapy (Figure 2A), when the survival of these patients was assessed from the date at which they were first diagnosed with a NET, rather than from the date of treatment, there was no significant difference between symptomatic responders (median 89±26.6 months) and non-responders (median 79±15.9 months) (Figure 3). This observation may reflect the fact that the symptomatic responders received ^131^I-MIBG treatment earlier in the course of their illness.

### Toxicity of ^131^I-MIBG therapy

The NCI toxicity criteria were used to assess the side effects of ^131^I-MIBG therapy (Table 3). Twelve (25%) patients developed haematological complications. There were seven (14.5%) cases of anaemia (all grade 1 or 2), three (6.2%) of thrombocytopaenia (one grade 1, one grade 2, one grade 3) and five (10.4%) of leucopenia (all grade 1 or 2). Nine patients showed disturbance of only one haematological parameter, whereas two developed both anaemia and thrombocytopaenia and one developed leucopenia and anaemia. All cases of myelosuppression resolved spontaneously. No patient developed renal failure, and three (6.3%) developed abnormal thyroid function tests (two hypothyroid, one hyperthyroid) despite thyroid blockade. One developed hypothyroidism after their first treatment, whereas the second patient was biochemically euthyroid after each of the first three treatments, but became hypothyroid after the fourth. Other symptomatic complications included nausea and vomiting, which occurred in nine (18.8%) patients and lethargy in seven (14.6%). Eleven (22.9%) patients required hospitalisation as a consequence of complications, mostly for monitoring of myelosuppression and for symptomatic relief of side effects. There was only one death within 30 days of treatment and this was due to tumour progression rather than due to treatment side effects. No patient experienced carcinoid crisis.

## DISCUSSION

In summary, we have demonstrated that ^131^I-MIBG therapy resulted in symptomatic improvement in more than half of a group of 48 patients with metastatic NETs. Patients who reported a symptomatic improvement also showed a 37-month increase in median survival from the date of first ^131^I-MIBG administration. Although some evidence of treatment-related toxicity was found in up to a third of patients, this toxicity was mild and self-limiting in most cases.

The proportion of patients experiencing symptomatic benefit from ^131^I-MIBG therapy (56.3%) was similar to that reported in previous studies (Safford *et al*, 2004; Sywak *et al*, 2004). As in one previous similar study (Safford *et al*, 2004), survival after ^131^I-MIBG therapy was best predicted by a subjective symptomatic response to treatment. Median survival was increased by 37 months in symptomatic responders compared to non-responders in this study, compared to 32 months in the previous study (Safford *et al*, 2004). As also previously demonstrated by Safford *et al* (2004), patients who demonstrated improvement in biochemical parameters (serum chromogranin A or 24 h urine 5HIAA) or decreased tumour burden on follow up radiological scans post-^131^I-MIBG therapy showed no statistically significant differences in survival. This finding may partly reflect the small sample size of patients who had complete biochemical and radiographic measurements available. Nonetheless, our data suggest that routine monitoring of biochemical and radiographic parameters post-^131^I-MIBG therapy is unlikely to provide useful information about an individual patient's prognosis.

Overall median survival in our cohort of patients (79±7.2 months) was similar to that reported in previous studies. In our study, although symptomatic responders to ^131^I-MIBG therapy showed significantly increased survival compared to non-responders when assessed from the date of first ^131^I-MIBG treatment, when survival of this group was assessed from the date of initial diagnosis with a NET, there was no statistically significant difference compared to those patients who did not demonstrate a symptomatic response to ^131^I-MIBG therapy. It is therefore possible that the responders to ^131^I-MIBG therapy were treated earlier in the course of their illness. It is not currently known whether a patient's response to radionuclide therapies alters during the course of disease progression. A randomised prospective trial in a less heterogeneous group of patients would be necessary to definitively determine whether ^131^I-MIBG therapy improves overall survival in patients with metastatic NETs.

Toxicity post-^131^I-MIBG therapy was not infrequent, occurring in 29.2% patients and leading to hospitalisation in 22.9%. Bone marrow suppression was observed most commonly, but in most cases this was mild (NCI grade 1 or 2) and resolved spontaneously without specific measures. There was one death within 30 days of treatment, but this was as a result of tumour progression rather than due to treatment side effects. Two patients developed hypothyroidism despite thyroid blockade.

Patients with NETs do benefit from a multidisciplinary approach to their care. All patients should be evaluated in a standardised manner using a range of biochemical assays as well as with anatomical and functional imaging techniques. Several treatment options are often possible for an individual patient at a particular stage in the course of his/her illness and the choice of treatment should therefore be discussed by the multidisciplinary team and with the patient concerned. Our study confirms previous observations that ^131^I-MIBG therapy is a generally safe and reasonably well-tolerated treatment option and confers symptomatic benefit in >50% patients with metastatic NETs. Symptomatic responders to ^131^I-MIBG therapy may have an improved prognosis. However, as responders in this study were often treated earlier in the course of their illness, current data do not confirm a significant increase in survival from the date of initial diagnosis. It therefore appears that the use of small-fractionated doses of ^131^I-MIBG probably does not affect the overall survival of patients with metastatic NETs, although therapy does appear to have a major effect on symptoms in many patients. The efficacy of larger doses of ^131^I-MIBG should therefore be evaluated, but as this is likely to be associated with an increased incidence of bone marrow toxicity risk/benefit analyses will have to be undertaken. Recently, other novel forms of radionuclide therapy have been developed. Direct comparisons of ^131^I-MIBG with other radionuclide therapies such as ^90^Y-DOTATOC (Bodei *et al*, 2004; Forrer *et al*, 2006; Frilling *et al*, 2006) should therefore be performed to determine the optimal treatment regimes for individual patients.

## Figures and Tables

**Table 1:** Patient demographics

**Characteristic**	**Total (%)**
Number of patients receiving ^131^I-MIBG	48 (100)
Female	27 (56.3)
Male	21 (43.8)
Mean age at time of diagnosis	57.6 (s.d. 10.9)
Mean age at time of first ^131^I-MIBG	61.7 (s.d. 10.0)
	
*Presenting symptoms*	
Diarrhoea	29 (60.4)
Flushing	28 (58.3)
Abdominal pain	20 (41.7)
Weight loss	15 (31.3)
Fatigue/tiredness	8 (16.7)
Bloating/distension	7 (14.5)
Breathlessness	6 (12.5)
Vomiting	5 (10.4)
Haemoptysis	3 (6.3)
Sweats	3 (6.3)
Loss of appetite	2 (4.2)
	
*Site of primary tumour*	
Small bowel	17 (33.3)
Lung	6 (12.5)
Pancreas	5 (10.4)
Caecum	3 (6.3)
Ascending colon	2 (4.2)
Liver	1 (2.1)
Stomach	1 (2.1)
Appendix	1 (2.1)
Unknown	12 (25.0)
	
*Presence of metastases*	48 (100)
Liver	44 (91.7)
Bone	4 (8.3)
Lymph node	13 (27.1)
	
*Previous surgery*	29 (60.4)
Small bowel resection	13 (27.1)
Right hemicolectomy	7 (14.6)
Other relevant resection	7 (14.6)
Shunt/bypass	7 (14.6)
Liver resection	6 (12.5)
	
*Other treatments*	
Somatostatin analogues	37 (77.1)
Chemotherapy	6 (12.5)
Interferon-*α*	2 (4.2)
Radiofrequency ablation	1 (2.1)
Hepatic chemoembolisation	7 (14.6)

**Table 2:** Clinical response to ^131^I-MIBG therapy

**Characteristic**	**Total (%)**
Biochemical markers (24 h urine 5-HIAA and/or serum chromogranin A) measured before and after ^131^I-MIBG treatment	29
>50% reduction of biochemical markers post-^131^I-MIBG therapy	11/29 (36.7)
Radiological investigations performed before and after ^131^I-MIBG treatment	40
>25% reduction in tumour size demonstrated on post-^131^I-MIBG scans	11/40 (27.5)
Symptomatic response to ^131^I-MIBG therapy	27/48 (56.2)

**Table 3:** Toxicity of ^131^I-MIBG therapy

		**NCI toxicity**			
**Characteristic**	**Number of patients (total 48)**	**1**	**2**	**3**	**4**
Hospitalisation	11	NA			
Thrombocytopenia	3	0	2	1	0
Anaemia	7	0	7	0	0
Leucopenia	5	0	5	0	0
Renal impairment	0	0	0	0	0
Thyroid abnormalities	3	NA			

Abbreviation: NA=not applicable.
